# Repeated measurements of serum urate and mortality: a prospective cohort study of 152,358 individuals over 8 years of follow-up

**DOI:** 10.1186/s13075-020-02173-4

**Published:** 2020-04-15

**Authors:** Shanshan Li, Liufu Cui, Jin Cheng, Rong Shu, Shuohua Chen, Uyen-Sa Nguyen, Devyani Misra, Shouling Wu, Xiang Gao

**Affiliations:** 1 Slone Epidemiology Center, Boston University, Boston University Medical Campus, 72 East Concord Street, L-7, Boston, MA 02118 USA; 2 Department of Rheumatology and Immunology, Kailuan General Hospital, Tangshan, China; 3 Department of Nutritional Sciences, The Pennsylvania State University, University Park, PA USA; 4 Health Care Center of Kailuan Group, Tangshan, China; 5 Department of Biostatistics and Epidemiology, University of North Texas Health Science Center School of Public Health, Fort Worth, TX USA; 6 Geriatrics and Rheumatology, Beth Israel Deaconess Medical Center, Boston, MA USA; 7 Department of Cardiology, Kailuan General Hospital, 57 Xinhua East Rd., Tangshan, 063000 China

**Keywords:** Serum urate, Mortality

## Abstract

**Background:**

Longitudinal evidence on change of serum urate level with mortality risk is limited as prior studies have a measurement of serum urate at a single time point. Further, the combined effect of serum urate and systemic inflammation on mortality is unknown.

**Methods:**

We conducted a prospective cohort study of 152,358 participants (122,045 men and 30,313 women) with repeated measurements of serum urate in 2006, 2008, 2010, and 2012 (107,751 participants had all four measurements of serum urate). We used the Cox proportional hazard model to examine the association between cumulative average and changes in serum urate with mortality. The combined effect of serum urate and systemic inflammation was determined by testing the interaction of serum urate and high-sensitive C-reactive protein (hs-CRP) in relation to mortality risk.

**Results:**

During a median follow-up of 8.7 (interquartile range 6.3–9.2) years, we identified 7564 all-cause deaths, 1763 CVD deaths, 1706 cancer deaths, and 1572 other deaths. We observed U-shaped relationships of cumulative average serum urate with all-cause mortality, cardiovascular mortality, and other mortalities. Compared with participants with stable serum urate, those with greater increases in serum urate had a 1.7-fold elevated mortality (hazard ratio (HR) = 1.66, 95% confidence interval (CI) = 1.49–1.84), and those with decreased serum urate had a 2-fold elevated mortality risk (HR = 2.14, 95% CI 1.93–2.37). Participants with both hyperuricemia and hs-CRP had 1.6 times higher mortality, compared with those with low serum urate and hs-CRP levels (HR = 1.56, 95% CI 1.37–1.76).

**Conclusions:**

We observed a U-shaped relationship of long-term cumulative average serum urate with all-cause mortality, cardiovascular mortality, and other mortalities. Compared with participants with relatively stable serum urate levels, a greater increase or decrease in serum urate was associated with elevated mortality. Participants with both hyperuricemia and high systemic inflammation had the greatest mortality risk compared with those with low serum urate and low hs-CRP levels.

## Significance

- Prior studies on the association between serum urate and mortality were limited by a single measurement of serum urate. To study the longitudinal relation, multiple measurements of serum urate levels are needed as serum uric acid may fluctuate over time.
- It is unknown whether a change (increase or decrease) in serum urate is associated with increased mortality.
- The combined effect of serum urate and inflammation on mortality is unknown.

## Innovation

- Both high and low serum urate concentrations averaged over four measurements were associated with elevated all-cause mortality, cardiovascular mortality, and other mortalities.
- Compared with participants with a relatively stable serum urate level, a greater increase or decrease in serum urate during the follow-up was associated with elevated mortality.
- Participants with both hyperuricemia and high systemic inflammation had a greater mortality risk compared with those with low serum urate and low systemic inflammation.

## Background

Serum urate is highly heritable [1] and has been shown to have antioxidant and pro-inflammatory properties [2–6]. Elevated serum urate induces inflammation; is associated with metabolic syndrome, carotid atherosclerosis, endothelia dysfunction, and oxidative stress; and has adverse effects on platelet adhesiveness and aggregation [6, 7]. However, whether serum urate is associated with higher mortality has long been under debate [8–28]. Evidence from previous observational studies [8–30] yields conflicting results and was limited by using only one-time measurement of serum urate and unobserved confounding [30, 31]. Thus, current evidence on the associations of serum urate with mortality needs to be interpreted with caution, given the wide heterogeneity in study designs, different categories of serum urate concentrations, and methodological limitations of only one measurement of serum urate [27, 28, 31]. The umbrella systemic review of serum urate with various health outcomes provided an overall view of the evidence on serum urate [28]. However, the quality of the evidence was assessed based on meta-analysis reports, instead of assessing the quality of each original research studies, thus still subjects to biases from the original individual studies. Mendelian randomization studies provided important evidence on a multitude of cardiovascular comorbidities associated with high serum urate levels [32], but evidence on the association between serum urate and mortality is limited [29].

Serum urate level changes over time. Whether changes in serum urate are associated with mortality, particularly among a healthy general population, remains unclear. Further, the combined effect of serum urate with systematic inflammation on risk for mortality is currently unknown. To the best of our knowledge, majority of the literature have a serum urate level measured at only one time point. Only three previous studies have serum urate measured at two time points, but none examined the changes in serum urate with mortality [23, 33, 34]. To the best of our knowledge, currently, no large prospective cohort study has repeated measurements of serum urate at multiple time points and is able to examine the long-term cumulative average of serum urate and changes in serum urate level in relation to all-cause and cause-specific mortality.

To address these knowledge gaps, we hypothesize that long-term elevated serum urate would be associated with higher mortality risk, and increases in serum urate would be associated with elevated mortality.

## Methods

### Study population

The Kailuan study, including the Kailuan studies I and II, is a large prospective cohort study that started in 2006 to investigate the risk factors for common non-communicable diseases in China [35, 36]. All residents in the Kailuan community (*n* = 155,418) aged 18 years or older and lived in Tangshan city were invited to participate in this study. Every 2 years, all participants underwent questionnaire assessments, physical examination, and laboratory tests in the hospitals. Trained nurses and physicians collected information regarding participants’ demographic, socioeconomic status, medical history, and lifestyle behavior information through a face-to-face survey. We excluded participants with missing information on serum urate and with cardiovascular disease or cancer at the study baseline. Our current analytic sample included 152,358 participants (122,045 men and 30,313 women) in the Kailuan studies I and II in the current study, who were followed from 2006 to 2015. Among these participants, 107,751 provided all four measurements of serum urate in 2006, 2008, 2010, and 2012. The study was approved by the Ethics Committee of the Kailuan Medical Group and the Brigham and Women’s Hospital, Boston. All participants gave their written informed consent.

### Measurement of serum urate

Study participants returned fasting blood samples in the morning after an 8- to 12-h overnight fast. Samples were transfused into vacuum tubes containing ethylene diamine tetra acetic acid (EDTA), collected and measured in 2006, 2008, 2010, and 2012. We calculated the cumulative average of repeated measurements of serum urate levels to represent long-term serum urate levels [37]. We calculated the changes between adjacent measurements and calculated a variable of per year change to represent changes in the serum urate over time. All calculations were based on available serum urate measurements. Because men have higher serum urate levels than women, sex-specific quintiles were calculated. Hyperuricemia is defined by cutoff values of > 7.0mg/dl for men and > 6.0 mg/dl for women.

### Outcome assessment

Mortality information was collected from provincial vital statistics offices [38]. Study clinicians reviewed death certificates and coded the main cause of death according to the criteria specified in the International Classification of Diseases, 10th Revision (ICD-10), as follows: cardiovascular (CVD)-specific mortality (codes I00–I09, I11, I13, I20–I51), cancer-specific mortality (codes C00 to C97), and all other causes.

### Measurement of high sensitive C-reactive protein

High sensitive C-reactive protein (hs-CRP) level was assessed by a commercial, high-sensitivity nephelometry assay (Cias Latex CRP-H, Kanto Chemical Co. Inc., Japan) at the central laboratory of Kailuan Hospital. For hs-CRP, the lower limit of detection was 0.1 mg/L, and intra- and inter-assay CVs were 6.53% and 4.78%, respectively. High C-reactive protein is defined as hs-CRP > 3 mg/L. Among our analytic sample, 141,426 participants have data on both serum urate and hs-CRP.

### Assessment of covariates

Information on demographic, socioeconomic status, medical history, and lifestyle information was collected using a self-report questionnaire. Height, weight, and blood pressure were assessed by trained nurses during the survey interviews. Total cholesterol, triglycerides, high-density lipoprotein cholesterol, low-density lipoprotein cholesterol, and creatinine were assessed using an auto-analyzer (Hitachi 747; Hitachi, Tokyo, Japan) at the central laboratory of Kailuan Hospital. We estimated the glomerular filtration rate using the Chronic Kidney Disease Epidemiology Collaboration creatinine equation [39, 40]. Fasting blood samples were collected in 2006, 2008, 2010, and 2012, after an 8- to 12-h overnight fast and transfused into vacuum tubes containing ethylene diamine tetra acetic acid (EDTA). Fasting blood glucose (FBG) level was measured using the Hitachi 747 auto-analyzer with CV < 2.0%. We further defined participants as having hypoglycemia (< 4.00 mmol/L [41]), normal FBG (4.00–5.59 mmol/L [42]), upper range of normal (5.60–6.09 mmol/L), impaired fasting glucose (6.10–6.99 mmol/L [43]), or diabetes (≥ 7.0 mmol/L). Participants with physician-diagnosed diabetes or use of hypoglycemic medications were assigned to the ≥ 7.00 mmol/L group.

### Statistical analyses

We used the Cox proportional hazard model to examine the association between long-term cumulative average serum urate level and changes in serum urate over time, with all-cause and cause-specific mortality. We defined changes in serum urate as quintiles, as well as a continuous variable representing per 10 mmol/L increment. Changes in serum urate were modeled as a time-varying exposure. To assess the possibility of reverse causation and examine “incident exposure” instead of “prevalent exposure,” we adjusted for baseline serum urate level in our multivariable-adjusted model [31]. To examine whether hyperuricemia confers elevated mortality beyond a gout diagnosis, we further excluded 500 participants with a self-reported history of gout. We also examined this association modeling serum urate using baseline measurement only, as well as model serum urate as a time-varying exposure. We tested the proportional hazards assumption by modeling multiplicative interaction terms between time and the primary exposure variable. We examined dose-response relationship using the Spline method.

To evaluate whether there is a combined effect of serum urate and inflammation on mortality, we further stratified analyses by hs-CRP level. Interaction on the multiplicative scale was tested by creating an interaction term between serum urate and hs-CRP in the multivariable model. Likelihood ratio tests were used to assess the significance of the interaction. We examined the multivariable association of hs-CRP levels for all-cause mortality, with and without additional adjustment for serum urate.

As undiagnosed major underlying conditions may influence serum urate level, we repeated our analyses excluding the first year of follow-up as sensitivity analyses. All analyses were performed using the SAS software, version 9.4 (Cary, NC).

## Results

During a median of 8.77 [interquartile range 6.3–9.2] years of follow-up, we identified 7564 death events, with 1763 CVD deaths and 1706 cancer deaths. Compared to participants in the lowest quintile of serum urate, those in the highest quintile had a higher education, were more likely to be current drinker, current smokers, consumed more sodium, had a higher BMI, and were more likely to use antihypertensive agent and lipid-lowering agent (Table 1).

**Table 1:** Baseline characteristics according to cumulative average serum urate concentrations

	Q1	Q2	Q3	Q4	Q5
*n*	30,475	30,526	30,455	30,461	30,441
Men, *n* (%)	24,221 (79.5)	24,288 (79.6)	24,164 (79.3)	24,252 (79.6)	24,185 (79.4)
Age, years	49.5 ± 13.4	49.7 ± 13.7	48.9 ± 14.3	48.2 ± 15.0	47.3 ± 17.1
Average income, *n* (%)					
< 500¥/month	5084 (16.7)	6218 (20.4)	6105 (20.0)	5763 (18.9)	4982 (16.4)
500–2999¥/month	20,643 (67.7)	19,717 (64.6)	19,595 (64.3)	19,361 (63.6)	18,582 (61.0)
≥ 3000¥/month	2507 (8.2)	2577 (8.4)	2678 (8.8)	2899 (9.5)	3250 (10.7)
Education, *n* (%)					
Illiteracy or elementary school	2648 (8.7)	2778 (9.1)	2645 (8.7)	2459 (8.1)	2295 (7.5)
Middle school	25,198 (82.7)	24,710 (80.9)	24,034 (78.9)	23,076 (75.8)	21,493 (70.6)
College/university	2116 (6.9)	2362 (7.7)	2887 (9.5)	3628 (11.9)	4387 (14.4)
Alcohol consumption status, *n* (%)					
Never	21,073 (69.1)	18,787 (61.5)	17,223 (56.6)	15,967 (52.4)	14,321 (47.0)
Past	722 (2.4)	842 (2.8)	904 (3.0)	849 (2.8)	838 (2.8)
Current	8225 (27.0)	10,322 (33.8)	11,559 (38.0)	12,471 (40.9)	13,210 (43.4)
Smoking status, *n* (%)					
Never	20,429 (67.0)	18,356 (60.1)	17,319 (56.9)	16,430 (53.9)	15,397 (50.6)
Past	1070 (3.5)	1320 (4.3)	1449 (4.8)	1622 (5.3)	1637 (5.4)
Current	8520 (28.0)	10,278 (33.7)	10,917 (35.8)	11,237 (36.9)	11,342 (37.3)
Sodium intake, *n* (%)					
≥ 10 g/day	2616 (8.6)	3047 (10.0)	3191 (10.5)	3483 (11.4)	3547 (11.7)
6–9 g/day	24,803 (81.4)	23,838 (78.1)	23,187 (76.1)	22,313 (73.3)	21,170 (69.5)
< 6 g/day	2581 (8.5)	3029 (9.9)	3257 (10.7)	3437 (11.3)	3539 (11.6)
Physical activity, *n* (%)					
Never	3699 (12.1)	4203 (13.8)	4434 (14.6)	4554 (15.0)	4889 (16.1)
1–2 times/week	22,908 (75.2)	21,601 (70.8)	20,663 (67.8)	20,044 (65.8)	18,776 (61.7)
3+ times/week	3389 (11.1)	4111 (13.5)	4537 (14.9)	4635 (15.2)	4593 (15.1)
Use of antihypertensive agent, *n* (%)	2896 (9.5)	3736 (12.2)	4373 (14.4)	5219 (17.1)	6542 (21.5)
Use of lipid-lowering agent, *n* (%)	264 (0.87)	386 (1.26)	494 (1.62)	564 (1.85)	716 (2.35)
Use of hypoglycemic agent, *n* (%)	1562 (5.13)	1496 (4.90)	1266 (4.16)	1167 (3.83)	1114 (3.66)
Use of aspirin, *n* (%)	185 (0.61)	268 (0.88)	321 (1.05)	336 (1.10)	372 (1.22)
Father’s CVD history, *n* (%)	1371 (4.50)	1786 (5.85)	1985 (6.52)	2032 (6.67)	2042 (6.71)
Mother’s CVD history, *n* (%)	972 (3.19)	1197 (3.92)	1323 (4.34)	1316 (4.32)	1400 (4.60)
FBG1, mmol/L	5.70 ± 1.81	5.61 ± 1.53	5.55 ± 1.39	5.53 ± 1.31	5.53 ± 1.26
BMI^1^, kg/m^2^	24.1 ± 3.0	24.4 ± 3.1	24.8 ± 3.1	25.3 ± 3.2	26.0 ± 3.4
eGFR^1^, mL/min/1.73 m^2^	85.8 ± 18.0	87.3 ± 17.9	88.4 ± 17.9	88.9 ± 18.2	88.6 ± 20.4
LDL-c^1^, mmol/L	2.50 ± 0.64	2.52 ± 0.68	2.53 ± 0.72	2.53 ± 0.76	2.53 ± 0.79
TG^1^, mmol/L	1.40 ± 1.00	1.44 ± 1.00	1.56 ± 1.16	1.74 ± 1.30	2.11 ± 1.62
SBP^1^, mmHg	129 ± 18	129 ± 18	130 ± 18	130 ± 18	132 ± 19
DBP^1^, mmHg	82.8 ± 9.7	83.0 ± 9.6	83.4 ± 9.8	84.0 ± 10.0	85.0 ± 10.3
hs-CRP^1,2^, mg/L	0.87 (1.26)	1.01 (1.36)	1.15 (1.48)	1.30 (1.67)	1.57 (2.01)

For continuous variables, mean ± SD

For categorical variables, *n* (%)

*Abbreviations*: *UA* serum urate, *FBG* fasting blood glucose, *BMI* body mass index, *eGFR* estimated glomerular filtration rate, *LDL-c* low-density lipoprotein cholesterol, *TG* triglycerides, *SBP* systolic blood pressure, *DBP* diastolic blood pressure, *hs-CRP* high sensitive C-reactive protein, *CVD* cardiovascular disease

^1^Cumulative average (see the “Methods” section)

^2^Present as median (interquartile range)

We observed a U-shaped relationship between long-term cumulative average serum urate level and all-cause mortality, CVD mortality, and other mortalities (Table 2, SFigure1). Comparing quintile 5 (mean serum urate = 326 (295–792) for women, 413 (372–890) for men; Stable 1) vs. quintile 3 (244 (231–258) for women, 304 (285–323) for men; Stable 1), higher serum urate was associated with 1.07 (95% confidence interval (CI) 0.99–1.15) times higher risk of all-cause mortality and 1.20 (95% CI 1.03–1.40) times higher risk of CVD mortality. The association with cancer mortality was inverse, with HRs comparing quintile 5 vs. quintile 3 of 0.73 (95% CI 0.62–0.86). Our results were consistent when we modeled serum urate using baseline measurement only and time-varying serum urate level (STable 3 and 4).

**Table 2:** The association between quintile of cumulative average serum urate and all-cause and cause-specific mortality

	Q1	Q2	Q3	Q4	Q5
All-cause mortality					
Population no. (cases)	30,475 (1713)	30,526 (1507)	30,455 (1462)	30,461 (1373)	30,441 (1509)
Incidence rate, per 1000 person-years	7.41	6.45	6.38	6.15	7.17
Age- and sex-adjusted HR	1.19 (1.11–1.27)	0.99 (0.92–1.06)	1 (ref)	0.97 (0.90–1.05)	1.11 (1.03–1.19)
Multiple adjusted HR*	1.12 (1.04–1.20)	0.97 (0.90–1.04)	1 (ref)	0.98 (0.91–1.05)	1.07 (0.99–1.15)
In 121,110 men HR*	1.11 (1.03–1.20)	0.97 (0.90–1.05)	1 (ref)	0.98 (0.91–1.06)	1.07 (0.99–1.16)
In 30,081 women HR*	1.35 (1.03–1.77)	0.98 (0.74–1.30)	1 (ref)	0.92 (0.70–1.21)	0.99 (0.77–1.29)
Cardiovascular mortality					
Population no. (cases)	30,475 (354)	30,526 (334)	30,455 (323)	30,461 (352)	30,441 (400)
Incidence rate, per 1000 person-years	1.53	1.43	1.41	1.57	1.89
Multiple adjusted HR*	1.06 (0.91–1.23)	0.98 (0.84–1.14)	1 (ref)	1.11 (0.96–1.30)	1.20 (1.03–1.40)
In 121,110 men HR*	1.04 (0.89–1.22)	0.97 (0.83–1.14)	1 (ref)	1.11 (0.95–1.30)	1.20 (1.02–1.40)
In 30,081 women HR*	1.55 (0.83–2.89)	1.08 (0.57–2.06)	1 (ref)	1.20 (0.66–2.17)	1.39 (0.80–2.42)
Cancer mortality					
Population no. (cases)	30,475 (397)	30,526 (351)	30,455 (386)	30,461 (314)	30,441 (258)
Incidence rate, per 1000 person-years	1.71	1.50	1.68	1.40	1.22
Multiple adjusted HR*	1.00 (0.87–1.16)	0.87 (0.75–1.00)	1 (ref)	0.85 (0.73–0.99)	0.73 (0.62–0.86)
In 121,110 men HR*	0.98 (0.85–1.14)	0.85 (0.73–0.99)	1 (ref)	0.85 (0.73–0.99)	0.70 (0.59–0.83)
In 30,081 women HR*	1.36 (0.81–2.30)	1.11 (0.65–1.88)	1 (ref)	0.92 (0.54–1.59)	0.99 (0.59–1.66)
Other mortalities					
Population no. (cases)	30,475 (367)	30,526 (328)	30,455 (285)	30,461 (267)	30,441 (325)
Incidence rate, per 1000 person-years	1.58	1.40	1.24	1.19	1.54
Multiple adjusted HR*	1.18 (1.01–1.38)	1.06 (0.91–1.25)	1 (ref)	1.00 (0.84–1.18)	1.22 (1.04–1.44)
In 121,110 men HR*	1.18 (1.00–1.39)	1.07 (0.91–1.26)	1 (ref)	1.00 (0.84–1.19)	1.21 (1.02–1.44)
In 30,081 women HR*	1.29 (0.72–2.34)	0.97 (0.52–1.79)	1 (ref)	0.90 (0.49–1.67)	1.19 (0.67–2.09)

*Model adjusted for age (years); sex; baseline serum urate (mmol/L); smoke status (current, past, or never); alcohol consumption status (current, past, or never); physical activity (never, sometimes, or active); average monthly income of each family member (< 500, 500–2999, or ≥ 3000¥); education (illiteracy/elementary school, middle school, or college/university); sodium intake (< 6.0, 6.0–9.9, or ≥ 10.0 g/day); father and mother’s cardiovascular disease history (yes or no); use of aspirin, antihypertensive, hypoglycemic, and lipid-lowering agents (yes/no for each); systolic blood pressure (quintile); diastolic blood pressure (quintile); fasting blood glucose (< 4.0, 4.0–5.5, 5.6–6.9, or ≥ 7 mmol/L); triglycerides (< 1.7, 1.7–2.2, 2.3–5.5, or ≥ 5.6 mmol/L); low-density lipoprotein cholesterol (< 1.80, 1.80–3.33, 3.34–4.91, or ≥ 4.92 mmol/L); body mass index (< 25.0, 25.0–29.9, or ≥ 30 kg/m^2^); high sensitive C-reactive protein (< 1, 1–2.9, or ≥ 3 mg/L); and estimated glomerular filtration rate (< 30, 30–59, 60–89, or ≥ 90 mL/min/1.73 m^2^)

Our results remained the same after we further excluded participants with gout (*n* = 501). Comparing quintile 5 vs. quintile 3 of serum urate, the multivariable-adjusted HR for all-cause mortality was 1.07 (95% CI 1.00–1.15).

Compared with the categories with minimum change (median (range) of changes in serum urate was 4 (1–8) for men and 4 (1–7) for women, STable 2), a greater increase in serum urate was associated with a 1.7-fold (95% CI 1.49–1.84) increased mortality, and a greater decrease in serum urate was associated with 2.0-fold (95% CI 1.93–2.37) increased mortality (Table 3). Similarly, this association was also observed for CVD mortality, cancer mortality, and all other mortalities. Results remained similar after we further excluded participants with gout. For annual changes in serum urate, the association with mortality was stronger compared to the results using cumulative average or baseline serum urate.

**Table 3:** The association between quintile of annually change in serum urate and all-cause and cause-specific mortality

	Q1	Q2	Q3	Q4	Q5
All-cause mortality					
Population no. (cases)	21,556 (1465)	21,603 (747)	21,494 (574)	21,548 (579)	21,550 (889)
Incidence rate, per 1000 person-years	8.23	4.01	3.06	3.13	5.16
Age- and sex-adjusted HR*	2.18 (1.98–2.41)	1.19 (1.07–1.33)	1 (ref)	1.10 (0.98–1.23)	1.92 (1.73–2.13)
Multiple adjusted HR*	2.14 (1.93–2.37)	1.20 (1.07–1.33)	1 (ref)	1.05 (0.93–1.17)	1.66 (1.49–1.84)
In 107,743 men HR*	2.15 (1.93–2.39)	1.20 (1.07–1.34)	1 (ref)	1.03 (0.91–1.16)	1.64 (1.47–1.83)
In 23,168 women HR*	2.16 (1.51–3.09)	1.21 (0.82–1.78)	1 (ref)	1.28 (0.87–1.88)	1.81 (1.26–2.61)
Cardiovascular mortality					
Population no. (cases)	21,556 (344)	21,603 (189)	21,494 (141)	21,548 (144)	21,550 (216)
Incidence rate, per 1000 person-years	1.93	1.01	0.75	0.78	1.25
Multiple adjusted HR*	2.20 (0.93–5.22)	2.53 (1.11–5.74)	1 (ref)	1.90 (0.78–4.62)	2.49 (1.03–6.01)
In 107,743 men HR*	1.80 (1.45–2.24)	1.23 (0.98–1.55)	1 (ref)	1.06 (0.83–1.35)	1.71 (1.36–2.15)
In 23,168 women HR*	1.52 (0.79–2.94)	0.96 (0.48–1.94)	1 (ref)	0.87 (0.42–1.83)	0.68 (0.31–1.51)
Cancer mortality					
Population no. (cases)	21,556 (356)	21,603 (176)	21,494 (147)	21,548 (136)	21,550 (208)
Incidence rate, per 1000 person-years	2.00	0.94	0.78	0.73	1.21
Multiple adjusted HR*	2.37 (1.93–2.90)	1.15 (0.92–1.43)	1 (ref)	0.97 (0.77–1.22)	1.57 (1.26–1.94)
In 107,743 men HR*	2.46 (1.99–3.04)	1.20 (0.96–1.51)	1 (ref)	0.93 (0.72–1.19)	1.48 (1.18–1.86)
In 23,168 women HR*	1.72 (0.86–3.47)	0.68 (0.29–1.57)	1 (ref)	1.43 (0.71–2.90)	2.65 (1.37–5.11)
Other mortality					
Population no. (cases)	21,556 (330)	21,603 (161)	21,494 (122)	21,548 (118)	21,550 (162)
Incidence rate, per 1000 person-years	1.85	0.86	0.65	0.64	0.94
Multiple adjusted HR*	2.34 (1.88–2.91)	1.21 (0.96–1.53)	1 (ref)	1.00 (0.78–1.29)	1.43 (1.12–1.81)
In 107,743 men HR*	2.22 (1.77–2.78)	1.11 (0.87–1.41)	1 (ref)	0.91 (0.70–1.18)	1.33 (1.04–1.70)
In 23,168 women HR*	6.98 (2.35–20.7)	4.94 (1.66–14.7)	1 (ref)	3.93 (1.28–12.0)	4.35 (1.42–13.3)

*Model adjusted for age (years); sex; baseline serum urate (mmol/L); smoke status (current, past, or never); alcohol consumption status (current, past, or never); physical activity (never, sometimes, or active); average monthly income of each family member (< 500, 500–2999, or ≥ 3000¥); education (illiteracy/elementary school, middle school, or college/university); sodium intake (< 6.0, 6.0–9.9, or ≥ 10.0 g/day); father and mother’s cardiovascular disease history (yes or no); use of aspirin, antihypertensive, hypoglycemic, and lipid-lowering agents (yes/no for each); systolic blood pressure (quintile); diastolic blood pressure (quintile); fasting blood glucose (< 4.0, 4.0–5.5, 5.6–6.9, or ≥ 7 mmol/L); triglycerides (< 1.7, 1.7–2.2, 2.3–5.5, or ≥ 5.6 mmol/L); low-density lipoprotein cholesterol(< 1.80, 1.80–3.33, 3.34–4.91, or ≥ 4.92 mmol/L); body mass index (< 25.0, 25.0–29.9, or 30 kg/m^2^); high sensitive C-reactive protein (< 1, 1–2.9, or ≥ 3 mg/L), and estimated glomerular filtration rate (< 30, 30–59, 60–89, or ≥ 90 mL/min/1.73 m^2^)

Participants with both hyperuricemia and high hs-CRP have elevated mortality risk. Compared to those with low serum urate and low hs-CRP level, those with high levels in both had a 1.6-fold increased risk for all-cause mortality and CVD mortality and a 1.7-fold increased risk for other mortalities. There was no elevated risk of cancer mortality (Table 4). Among participants with low hs-CRP, those who also have a high serum urate level have significantly higher all-cause mortality, CVD mortality, and other mortalities compared with participants with low serum urate levels. We did not observe a significant interaction between serum urate and hs-CRP. Comparing participants with hs-CRP level > 3 mg/L vs. < 1 mg/L, multivariate-adjusted HR was 1.19 (95% CI 1.13–1.25, Stable 5). Further adjustment for serum urate did not change the observed association (HR = 1.18, 95% CI 1.13–1.24, Stable 5). Multivariable-adjusted HR of hyperuricemia (yes vs. no) for all-cause mortality was 1.26 (95% CI 1.13–1.42).

**Table 4:** The combined effect of serum urate and high sensitive C-reactive protein with all-cause and cause-specific mortality

	Low hs-CRP low serum urate	Low hs-CRP hyperuricemia	High hs-CRP low serum urate	High hs-CRP hyperuricemia	P for interaction
All-cause mortality					
Population no. (cases)	110,643 (4717)	7577 (386)	20,381 (2023)	2825 (275)	
Incidence rate, per 1000 person-years	5.50	7.31	13.5	14.4	
Age- and sex-adjusted HR	1 (ref)	1.20 (1.08–1.33)	1.65 (1.57–1.74)	1.82 (1.61–2.06)	0.29
Multiple adjusted HR*	1 (ref)	1.16 (1.04–1.29)	1.49 (1.41–1.57)	1.56 (1.37–1.76)	0.20
In 121,110 men HR*	1 (ref)	1.16 (1.04–1.30)	1.51 (1.42–1.59)	1.51 (1.32–1.73)	0.09
In 30,081 women HR*	1 (ref)	0.95 (0.63–1.43)	1.25 (1.02–1.54)	1.79 (1.23–2.62)	0.14
Cardiovascular mortality					
Population no. (cases)	110,643 (1055)	7577 (109)	20,381 (481)	2825 (74)	
Incidence rate, per 1000 person-years	1.23	2.06	3.21	3.88	
Multiple adjusted HR*	1 (ref)	1.36 (1.11–1.67)	1.44 (1.28–1.61)	1.55 (1.21–1.97)	0.15
In 121,110 men HR*	1 (ref)	1.39 (1.12–1.71)	1.43 (1.27–1.61)	1.46 (1.12–1.90)	0.07
In 30,081 women HR*	1 (ref)	1.05 (0.47–2.36)	1.40 (0.92–2.13)	2.49 (1.25–4.97)	0.32
Cancer mortality					
Population no. (cases)	110,643 (1148)	7577 (57)	20,381 (437)	2825 (41)	
Incidence rate, per 1000 person-years	1.34	1.08	2.91	2.15	
Multiple adjusted HR*	1 (ref)	0.75 (0.57–0.98)	1.54 (1.38–1.73)	1.17 (0.85–1.60)	0.96
In 121,110 men HR*	1 (ref)	0.77 (0.58–1.02)	1.55 (1.38–1.75)	1.05 (0.74–1.50)	0.58
In 30,081 women HR*	1 (ref)	0.43 (0.13–1.40)	1.32 (0.89–1.97)	1.91 (0.87–4.20)	0.08
Other mortalities					
Population no. (cases)	110,643 (963)	7577 (94)	20,381 (417)	2825 (58)	
Incidence rate, per 1000 person-years	1.12	1.78	2.78	3.04	
Multiple adjusted HR*	1 (ref)	1.48 (1.19–1.84)	1.53 (1.35–1.72)	1.77 (1.34–2.33)	0.16
In 121,110 men HR*	1 (ref)	1.42 (1.13–1.79)	1.54 (1.36–1.75)	1.82 (1.37–2.42)	0.32
In 30,081 women HR*	1(ref)	1.84 (0.88–3.85)	1.33 (0.85–2.09)	1.30 (0.44–3.81)	0.32

Hyperuricemia is defined by a cutoff values of > 7.0mg/dl for men and > 6.0 mg/dl for women

High C-reactive protein is defined as hs-CRP > 3 mg/L.

*Model adjusted for age (year); sex; smoke status (current, past, or never); alcohol consumption status (current, past, or never); physical activity (never, sometimes, or active); average monthly income of each family member (< 500, 500–2999, or ≥ 3000¥); education (illiteracy/elementary school, middle school, or college/university); sodium intake (< 6.0, 6.0–9.9, or ≥ 10.0 g/day); father and mother’s cardiovascular disease history (yes or no); use of aspirin, antihypertensive, hypoglycemic, and lipid-lowering agents (yes/no for each); systolic blood pressure (quintile); diastolic blood pressure (quintile); fasting blood glucose (< 4.0, 4.0–5.5, 5.6–6.9, or ≥ 7 mmol/L); triglycerides (< 1.7, 1.7–2.2, 2.3–5.5, or ≥ 5.6 mmol/L); low-density lipoprotein cholesterol (< 1.80, 1.80–3.33, 3.34–4.91, or ≥ 4.92 mmol/L); body mass index (< 25.0, 25.0–29.9, or ≥ 30 kg/m^2^); and estimated glomerular filtration rate (< 30, 30–59, 60–89, or ≥ 90 mL/min/1.73 m^2^)

Our results remained similar, and conclusions remained the same when we repeated our analyses after excluding the first year of follow-up as sensitivity analyses.

## Discussion

In this study of 152,358 participants with 8.7 years of follow-up, we found that higher cumulative average serum urate was associated with elevated all-cause mortality, CVD mortality, and other mortalities. Greater annual increases or decreases in serum urate were both associated with elevated mortality risk. Participants with both hyperuricemia and high hs-CRP had 1.6 times higher all-cause mortality and 2.5 times higher cardiovascular mortality, compared with those with low serum urate and low hs-CRP level.

The association between serum urate and mortality has been long debated with conflicting results [8–30]. Whether the elevated mortality risk among people with gout is due to the adverse effects of hyperuricemia or comorbidities and lifestyle factors that travel together with hyperuricemia is a question needed to be clarified. Prior studies were mainly focused on patients with specific conditions. Some suggested a J- or U-shaped relationship [9–15] or a strong positive relationship, while others suggested a null association [16–23] or inverse association [24]. Direct evidence for healthy individuals is limited [44, 45]. Further, serum urate varies over time; thus, prior studies evaluating serum urate were limited with only one single measurement of serum urate and were unable to examine the longitudinal association between long-term serum urate and mortality [31]. One single measurement of serum urate was also subject to potential regression dilution bias and reverse causation issue [31]. Among the general population, evidence from cohort studies were mostly with only one measurement of serum urate [9, 16, 28, 46–48], with sparse evidence on cumulative average [23, 34] or changes in serum urate [34]. Thus, the long-term cumulative effects of serum urate with mortality risk are unclear.

In our study, we aimed to address these knowledge gaps and methodological limitations by leveraging the unique data with four repeated measurements of serum urate with long-term follow-up, accounting for covariates on BMI, alcohol intake, diet, blood pressure, kidney function, fasting glucose, and other potential confounders. Serum urate levels could be affected by many lifestyle factors, such as alcohol intake and BMI. It is well-known that these factors are related to mortality risk. We adjusted for these factors in our multivariable model and observed a consistent pattern of a U-shaped relationship between cumulative average serum urate and all-cause mortality, CVD mortality, and other mortalities. This relationship is consistent with previous findings from studies of Asian general population cohorts [44, 45]. Results from our current study further showed that the association remained significant and independent of kidney function [21, 49]. In our study, in the analysis of serum urate measured at baseline in relation to all-cause mortality, the multivariate HRs for Q1, Q2, Q4, and Q5 h/week, relative to Q3, were 1.03 (95% CI 0.96–1.11), 1.00 (95% CI 0.93–1.08), 1.03 (95% CI 0.96–1.11), and 1.10 (95% CI 1.02–1.18), respectively. These HRs were similar to those shown in Table 2, which were derived with cumulative average serum urate across multiple measurements. Similar patterns were observed for cause-specific mortality. The results of our study showed that higher serum urate, either single measurement of serum urate or multiple measurements of serum urate, is associated with higher mortality. HRs were similar from the analysis using single vs. multiple measurements of serum urate. However, interpretations of these results were different. Single measurement of serum urate is a fixed exposure, representing prevalent exposure, while multiple measurements of serum urate represent long-term cumulative average [31].

With regard to serum urate with cancer mortality, results from our study indicate an inverse association between long-term cumulative average serum urate and cancer mortality. An annual decrease or increase in serum urate was associated with a 2.3- and 1.6-fold increased risk of cancer mortality, respectively. Prior studies on serum urate with cancer mortality have yielded conflicting results [14, 29, 44, 50–54], with some reporting lower risk [51, 52]and some reporting increased risk [14, 29, 44, 53, 54] of cancer mortality. Potential explanations for these inconsistent findings include not being able to examine both low and high serum urate levels, incomplete covariate adjustment, and inability to examine changes in serum urate with cancer mortality.

In addition, serum urate levels could be affected by many lifestyle factors, such as alcohol intake and BMI. The time-varying confounding by these factors has not been appropriately accounted for in prior studies. Our study was the first study that was able to model the time-varying effects of serum urate, as well as long-term cumulative average effects of serum urate. More importantly, this knowledge could help shape clinical decision-making and future study design as focus shifts from a single measurement of serum urate to multiple measurements and measuring changes in serum urate over time, and prevention of risk factors development. Also, determining the combined impacts of serum urate and hs-CRP could significantly improve preventive and treatment efforts.

The precise interrelationship between levels of serum urate and the role of inflammation in increasing mortality risk remains rudimentary. Serum urate correlates with many cardiometabolic traits, including hypertension, dyslipidemia, type 2 diabetes, cardiovascular disease, and metabolic syndrome [6, 7]. High serum urate activates the renin-angiontensin system, promotes inflammation, accelerates LDL oxidation, reduces the nitric oxide, and induces endothelial dysfunction [2–5]. Serum urate is also a potent antioxidant and contributes to more than 50% of human plasma antioxidant capacity [2, 6]. How a combination of varying degrees of inflammation and serum urate confers differential risk of systemic consequences is not clear. In our study, we found that the mortality risk of both high serum urate and hs-CRP is substantially higher than that of high hs-CRP only. Participants with both hyperuricemia and high hs-CRP had 1.6 times higher all-cause mortality, compared with those with low serum urate and low hs-CRP level. The multivariate-adjusted HR of hs-CRP for all-cause mortality was 1.19 (95% CI 1.13–1.25) and did not change after further adjustment for serum urate. Future studies, particularly from other races/ethnicities, are needed to confirm this observation.

Our study’s strengths and limitations warrant discussion. Our study has repeated measurements of serum urate concentration, large sample size, detailed covariates information including kidney function, fasting glucose and lifestyle factors, medical conditions, medication use, and cause-specific information about death events. We were also able to examine the interaction between serum urate and hs-CRP, which provides novel insights regarding the complex interactions between serum urate and inflammatory markers. We have detailed information on the changes in serum urate, which improve our understanding of whether increase or decrease serum urate would modify mortality risk, a question prior studies could not address.

Our study also has some limitations. We do not have information regarding commonly used urate-lowering management strategies or medication use such as allopurinol. How these treatment strategies could potentially lower mortality for hyperuricemia populations is relatively under-studied. However, our results did not materially change after we excluded participants with gout. Second, only hs-CRP as the inflammatory marker was available in the current study. Future studies are needed to further examine the combined effects of serum urate with other inflammatory markers. Even though we have extensive covariate control in our study, we may still have some residual and unmeasured confounding. Further, the serum urate level in our study population was relatively low compared with the Western population; thus, our results may not generalizable to Western populations. Future studies that elucidate the underlying biological mechanisms are needed.

## Conclusions

In conclusion, we observed a U-shaped relationship between long-term cumulative average serum urate level and all-cause and cause-specific mortality. Compared with participants with relatively stable urate levels, greater increases or decreases in serum urate were both associated with elevated all-cause and cause-specific mortality. Participants with both hyperuricemia and systemic inflammation had the greatest mortality risk compared with those with both low serum urate and hs-CRP levels.

## Supplementary information

**Additional file 1: Table S1.** The median and range of serum urate for each quintile.

**Additional file 2: Table S2.** Median change (range) of serum urate per year.

**Additional file 3: Table S3.** The association between baseline serum urate, all-cause and cause-specific mortality.

**Additional file 4: Table S4.** The association between time varying serum urate, all-cause and cause-specific mortality.

**Additional file 5: Table S5.** Hazard ratio and 95% confidence interval for the association between high sensitivity C-reactive protein and all-cause mortality.

**Additional file 6: Figure S1.** Dose response curve of serum urate with all-cause mortality.

## Data Availability

The data generated by our research could be made available upon request to the corresponding authors.
