# Supplementary material for: Repeated measurements of serum urate and mortality: a prospective cohort study of 152,358 individuals over 8 years of follow-up
Source: Arthritis Res Ther. 2020 Apr 15;22:84. doi: 10.1186/s13075-020-02173-4 (PMC7160947; doi:10.1186/s13075-020-02173-4)
Supplement: Supplementary file 1 — Additional file 1: Table S1. The median and range of serum urate for each quintile. [file 13075_2020_2173_MOESM1_ESM.docx]

**STable 1. The median and range of serum urate for each quintile**

|  | **Q1** | **Q2** | **Q3** | **Q4** | **Q5** |
| --- | --- | --- | --- | --- | --- |
|  | **Cumulative average serum urate** | | | | |
| **Women** | 185(24-204) | 218(204-231) | 244(231-258) | 274(258-294) | 326(295-792) |
| **Men** | 221(12-246) | 266(246-285) | 304(285-323) | 345(323-372) | 413(372-890) |
|  | **Baseline serum urate** | | | | |
| **Women** | 163(21-189) | 206(189-221) | 236(221-253) | 273(253-295) | 333(295-817) |
| **Men** | 208(12-236) | 258(236-278) | 298(278-319) | 343(319-372) | 416(372-1457) |
