# Supplementary material for: Repeated measurements of serum urate and mortality: a prospective cohort study of 152,358 individuals over 8 years of follow-up
Source: Arthritis Res Ther. 2020 Apr 15;22:84. doi: 10.1186/s13075-020-02173-4 (PMC7160947; doi:10.1186/s13075-020-02173-4)
Supplement: Supplementary file 2 — Additional file 2: Table S2. Median change (range) of serum urate per year. [file 13075_2020_2173_MOESM2_ESM.docx]

**STable 2. Median change (range) of serum urate per year**

|  | Q1 | Q2 | Q3 | Q4 | Q5 |
| --- | --- | --- | --- | --- | --- |
| Men | -20(-230 to -10) | -4(-9 to 0) | 4(1 to 8) | 12(9 to 18) | 28(19 to 267) |
| Women | -17(-300 to -9) | -4(-8 to 0) | 4(1 to 7) | 12(8 to 17) | 27(18 to 265) |
