# Supplementary material for: Repeated measurements of serum urate and mortality: a prospective cohort study of 152,358 individuals over 8 years of follow-up
Source: Arthritis Res Ther. 2020 Apr 15;22:84. doi: 10.1186/s13075-020-02173-4 (PMC7160947; doi:10.1186/s13075-020-02173-4)
Supplement: Supplementary file 3 — Additional file 3: Table S3. The association between baseline serum urate, all-cause and cause-specific mortality. [file 13075_2020_2173_MOESM3_ESM.docx]

**STable 3. The association between baseline serum urate, all-cause and cause-specific mortality**

|  | **Q1** | **Q2** | **Q3** | **Q4** | **Q5** |
| --- | --- | --- | --- | --- | --- |
| **All-cause mortality** |  |  |  |  |  |
| Population # (case) | 30648(1551) | 30291(1477) | 30313(1446) | 30745(1445) | 30361(1645) |
| Incidence rate, per 1000 person-year | 6.57 | 6.43 | 6.38 | 6.45 | 7.78 |
| Age and sex adjusted HR | 1.09(1.01-1.17) | 1.01(0.94-1.08) | 1(ref) | 1.00(0.93-1.08) | 1.11(1.03-1.19) |
| Multiple adjusted HR^*^ | 1.03(0.96-1.11) | 1.00(0.93-1.08) | 1(ref) | 1.03(0.96-1.11) | 1.10(1.02-1.18) |
| In 121,110 men HR* | 1.04(0.97-1.12) | 0.99(0.92-1.07) | 1(ref) | 1.05(0.97-1.13) | 1.11(1.03-1.19) |
| In 30,081 women HR* | 0.90(0.68-1.20) | 1.20(0.92-1.57) | 1(ref) | 0.84(0.64-1.11) | 1.01(0.79-1.30) |
|  |  |  |  |  |  |
| **Cardiovascular mortality** |  |  |  |  |  |
| Population # (case) | 30648(333) | 30291(324) | 30313(305) | 30745(371) | 30361(430) |
| Incidence rate, per 1000 person-year | 1.41 | 1.41 | 1.34 | 1.65 | 2.03 |
| Multiple adjusted HR^*^ | 1.06(0.91-1.24) | 1.06(0.90-1.24) | 1(ref) | 1.24(1.07-1.45) | 1.29(1.11-1.50) |
| In 121,110 men HR* | 1.09(0.93-1.28) | 1.05(0.89-1.24) | 1(ref) | 1.28(1.10-1.50) | 1.28(1.09-1.50) |
| In 30,081 women HR* | 0.73(0.37-1.45) | 1.24(0.69-2.22) | 1(ref) | 0.77(0.42-1.42) | 1.49(0.90-2.47) |
|  |  |  |  |  |  |
| **Cancer mortality** |  |  |  |  |  |
| Population # (case) | 30648(338) | 30291(352) | 30313(386) | 30745(324) | 30361(306) |
| Incidence rate, per 1000 person-year | 1.43 | 1.53 | 1.70 | 1.44 | 1.44 |
| Multiple adjusted HR^*^ | 0.83(0.72-0.97) | 0.88(0.77-1.02) | 1(ref) | 0.87(0.75-1.00) | 0.82(0.70-0.96) |
| In 121,110 men HR* | 0.85(0.73-0.99) | 0.86(0.74-1.00) | 1(ref) | 0.88(0.75-1.02) | 0.82(0.69-0.96) |
| In 30,081 women HR* | 0.64(0.37-1.13) | 1.23(0.77-1.98) | 1(ref) | 0.77(0.46-1.30) | 0.80(0.49-1.30) |
|  |  |  |  |  |  |
| **Other mortality** |  |  |  |  |  |
| Population # (case) | 30648(335) | 30291(293) | 30313(291) | 30745(295) | 30361(358) |
| Incidence rate, per 1000 person-year | 1.42 | 1.27 | 1.28 | 1.31 | 1.69 |
| Multiple adjusted HR^*^ | 1.06(0.91-1.24) | 0.97(0.83-1.14) | 1(ref) | 1.06(0.90-1.25) | 1.23(1.05-1.44) |
| In 121,110 men HR* | 1.06(0.90-1.25) | 0.95(0.80-1.12) | 1(ref) | 1.07(0.90-1.27) | 1.21(1.03-1.43) |
| In 30,081 women HR* | 1.04(0.56-1.94) | 1.29(0.72-2.33) | 1(ref) | 0.85(0.46-1.60) | 1.30(0.75-2.27) |

^*^Model adjusted for age (year), sex, smoke status (current, past, or never), alcohol consumption status (current, past, or never), physical activity (never, sometimes, or active), average monthly income of each family member (<[500, 500-2999](callto:500,%20500-2999), or ≥3000¥), education (illiteracy/elementary school, middle school, or college/university), sodium intake (<6.0, 6.0-9.9, or ≥10.0 gram/day), father and mother’s cardiovascular disease history (yes or no), use of aspirin, antihypertensive, hypoglycemic, and lipid-lowering agents (yes/no for each), systolic blood pressure (quintile), diastolic blood pressure (quintile), fasting blood glucose (<4.0, 4.0-5.5,5.6-6.9, or ≥7 mmol/L), triglycerides(<1.7, 1.7-2.2, 2.3-5.5, or ≥5.6 mmol/L), low-density lipoprotein cholesterol (<1.80, [1.80](callto:4.92,%203.34-4.91,%201.81)-3.33, 3.34-4.91, or ≥ 4.92 mmol/L), body mass index (<25.0, 25.0-29.9, or ≥ 30 Kg/m^2^), high sensitive C-reactive protein (<1, 1-2.9, or ≥3mg/L), and estimated glomerular filtration rate (<30, 30-59, 60-89, or ≥90 mL/min/1.73m^2^). Quintiles are sex-specific quintiles.
