# Supplementary material for: Repeated measurements of serum urate and mortality: a prospective cohort study of 152,358 individuals over 8 years of follow-up
Source: Arthritis Res Ther. 2020 Apr 15;22:84. doi: 10.1186/s13075-020-02173-4 (PMC7160947; doi:10.1186/s13075-020-02173-4)
Supplement: Supplementary file 4 — Additional file 4: Table S4. The association between time varying serum urate, all-cause and cause-specific mortality. [file 13075_2020_2173_MOESM4_ESM.docx]

**STable 4. The association between time varying serum urate, all-cause and cause-specific mortality**

|  | **Q1** | **Q2** | **Q3** | **Q4** | **Q5** |
| --- | --- | --- | --- | --- | --- |
| **All-cause mortality** |  |  |  |  |  |
| Population # (case) | 30469(1440) | 30311(1460) | 30631(1488) | 30482(1554) | 30465(1622) |
| Incidence rate, per 1000 person-year | 6.23 | 6.23 | 6.47 | 6.96 | 7.77 |
| Multiple adjusted HR^*^ | 1.20(1.12-1.29) | 1.02(0.95-1.10) | 1(ref) | 1.03(0.96-1.11) | 1.09(1.01-1.17) |
| In 121,110 men HR* | 1.21(1.12-1.30) | 1.02(0.95-1.10) | 1(ref) | 1.02(0.95-1.10) | 1.08(1.01-1.17) |
| In 30,081 women HR* | 1.28(0.99-1.64) | 1.04(0.80-1.36) | 1(ref) | 1.14(0.84-1.55) | 1.29(0.92-1.80) |
|  |  |  |  |  |  |
| **Cardiovascular mortality** |  |  |  |  |  |
| Population # (case) | 30469(300) | 30311(312) | 30631(324) | 30482(395) | 30465(432) |
| Incidence rate, per 1000 person-year | 1.30 | 1.33 | 1.41 | 1.77 | 2.06 |
| Multiple adjusted HR^*^ | 1.17(0.99-1.37) | 1.01(0.87-1.18) | 1(ref) | 1.19(1.03-1.39) | 1.27(1.10-1.48) |
| In 121,110 men HR* | 1.20(1.02-1.42) | 1.05(0.89-1.23) | 1(ref) | 1.21(1.04-1.41) | 1.29(1.11-1.51) |
| In 30,081 women HR* | 0.93(0.57-1.54) | 0.71(0.41-1.22) | 1(ref) | 1.00(0.55-1.81) | 1.27(0.68-2.37) |
|  |  |  |  |  |  |
| **Cancer mortality** |  |  |  |  |  |
| Population # (case) | 30469(337) | 30311(344) | 30631(386) | 30482(368) | 30465(271) |
| Incidence rate, per 1000 person-year | 1.46 | 1.46 | 1.67 | 1.64 | 1.29 |
| Multiple adjusted HR^*^ | 1.08(0.93-1.25) | 0.92(0.80-1.07) | 1(ref) | 0.94(0.82-1.09) | 0.74(0.63-0.86) |
| In 121,110 men HR* | 1.07(0.91-1.26) | 0.91(0.78-1.06) | 1(ref) | 0.92(0.79-1.07) | 0.73(0.62-0.86) |
| In 30,081 women HR* | 1.37(0.84-2.25) | 1.15(0.68-1.96) | 1(ref) | 1.36(0.75-2.47) | 1.06(0.50-2.23) |
|  |  |  |  |  |  |
| **Other mortality** |  |  |  |  |  |
| Population # (case) | 30469(310) | 30311(299) | 30631(319) | 30482(303) | 30465(341) |
| Incidence rate, per 1000 person-year | 1.34 | 1.27 | 1.38 | 1.35 | 1.63 |
| Multiple adjusted HR^*^ | 1.15(0.98-1.35) | 0.96(0.82-1.12) | 1(ref) | 0.96(0.82-1.12) | 1.11(0.95-1.30) |
| In 121,110 men HR* | 1.17(0.99-1.38) | 0.96(0.82-1.13) | 1(ref) | 0.94(0.80-1.11) | 1.09(0.93-1.28) |
| In 30,081 women HR* | 1.12(0.65-1.92) | 0.92(0.51-1.65) | 1(ref) | 1.08(0.55-2.13) | 1.61(0.78-3.30) |

^*^Model adjusted for age (year), sex, baseline serum urate (mmol/L), smoke status (current, past, or never), alcohol consumption status (current, past, or never), physical activity (never, sometimes, or active), average monthly income of each family member (<[500, 500-2999](callto:500,%20500-2999), or ≥3000¥), education (illiteracy/elementary school, middle school, or college/university), sodium intake (<6.0, 6.0-9.9, or ≥10.0 gram/day), father and mother’s cardiovascular disease history (yes or no), use of aspirin, antihypertensive, hypoglycemic, and lipid-lowering agents (yes/no for each), systolic blood pressure (quintile), diastolic blood pressure (quintile), fasting blood glucose (<4.0, 4.0-5.5,5.6-6.9, or ≥7 mmol/L), triglycerides (<1.7, 1.7-2.2, 2.3-5.5, or ≥5.6 mmol/L), low-density lipoprotein cholesterol (<1.80, [1.80](callto:4.92,%203.34-4.91,%201.81)-3.33, 3.34-4.91, or 4.92,≥mmol/L), body mass index (<25.0, 25.0-29.9, or ≥30Kg/m^2^), high sensitive C-reactive protein (<1, 1-2.9, or≥3mg/L), and estimated glomerular filtration rate (<30, 30-59, 60-89, or ≥90 mL/min/1.73m^2^).
