# Supplementary material for: Repeated measurements of serum urate and mortality: a prospective cohort study of 152,358 individuals over 8 years of follow-up
Source: Arthritis Res Ther. 2020 Apr 15;22:84. doi: 10.1186/s13075-020-02173-4 (PMC7160947; doi:10.1186/s13075-020-02173-4)
Supplement: Supplementary file 5 — Additional file 5: Table S5. Hazard ratio and 95% confidence interval for the association between high sensitivity C-reactive protein and all-cause mortality. [file 13075_2020_2173_MOESM5_ESM.docx]

**Stable 5. Hazard ratio and 95% confidence interval for the association between high sensitivity C-reactive protein and all-cause mortality.**

|  | **CRP <1mg/L** | **CRP 1-2.9 mg/L** | **CRP ≥3 mg/L** | **Log-CRP** | **P -trend** |
| --- | --- | --- | --- | --- | --- |
| Multivariate model | 1.00 (ref) | 1.11 (1.06, 1.16) | 1.19 (1.13, 1.25) | 1.06 (1.04, 1.07) | <0.001 |
| Multivariate + serum urate | 1.00 (ref) | 1.10 (1.05, 1.16) | 1.18 (1.13, 1.24) | 1.06 (1.04, 1.07) | <0.001 |

Multivariate model adjusted for age (year), sex, smoke status (current, past, or never), alcohol consumption status (current, past, or never), physical activity (never, sometimes, or active), average monthly income of each family member (<500, 500-2999, or ≥3000¥), education (illiteracy/elementary school, middle school, or college/university), sodium intake (<6.0, 6.0-9.9, or ≥10.0 gram/day), father and mother’s cardiovascular disease history (yes or no), use of antihypertensive, hypoglycemic, and lipid-lowering agents (yes/no for each), systolic blood pressure (quintile), diastolic blood pressure (quintile), fasting blood glucose (<4.0, 4.0-5.5,5.6-6.9, or ≥7 mmol/L), triglycerides (<1.7, 1.7-2.2, 2.3-5.5, or ≥5.6 mmol/L), low-density lipoprotein cholesterol (<1.80, 1.80-3.33, 3.34-4.91, or ≥4.92 mmol/L), body mass index (<25.0, 25.0-29.9, or ≥30 Kg/m^2,^ and estimated glomerular filtration rate (<30, 30-59, 60-89, or ≥90 mL/min/1.73m^2^).
