# Supplementary material for: Repeated measurements of serum urate and mortality: a prospective cohort study of 152,358 individuals over 8 years of follow-up
Source: Arthritis Res Ther. 2020 Apr 15;22:84. doi: 10.1186/s13075-020-02173-4 (PMC7160947; doi:10.1186/s13075-020-02173-4)
Supplement: Supplementary file 6 [file 13075_2020_2173_MOESM6_ESM.docx]

SFigure 1. Dose response curve of serum urate with all-cause mortality

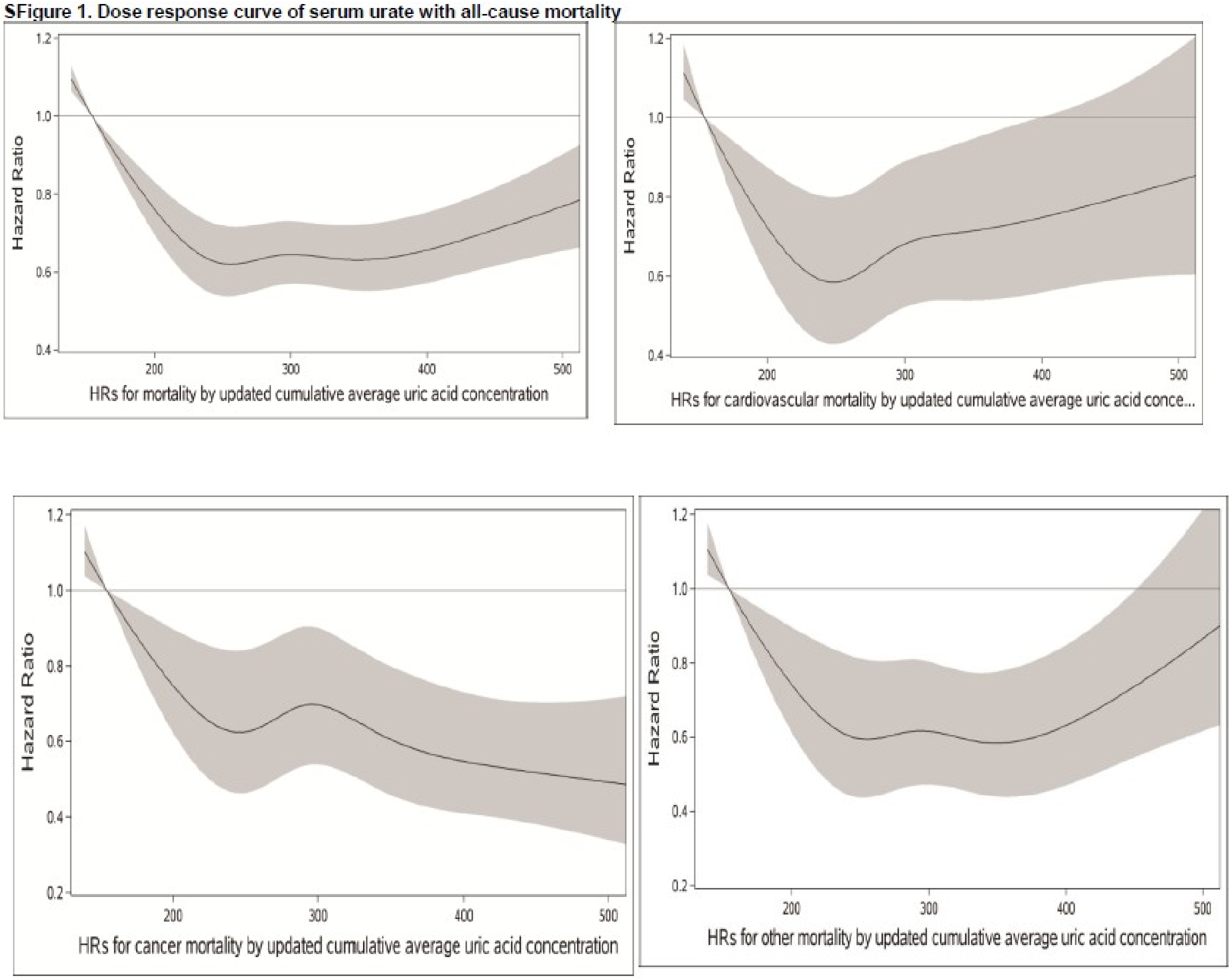

Figure legend:
X axis Hazard ratios and its 95% confidence interval
Y axis cumulative average of serum urate level
